# Poly(1-naphthylamine)-Reinforced Chitosan Films for Smart Packaging: Enhanced Mechanical, Morphological, and Antibacterial Properties

**DOI:** 10.1021/acs.langmuir.5c04826

**Published:** 2025-11-12

**Authors:** Mary Taylor, Jayla Jenkins, Cristian Rodriguez, Audrey Adcock, Liju Yang, Mohammad Mohiuddin, Ufana Riaz

**Affiliations:** 1 Julius L. Chambers Biomedical/Biotechnology Research Institute (BBRI), 3066 North Carolina Central University, 1801 Fayetteville St., Durham, North Carolina 27707, United States; 2 School of Packaging, 115974 Michigan State University, 110 Packaging Building 448 Wilson Road, East Lansing, Michigan 48824-1223, United States; 3 Biomanufacturing Research Institute and Technology Enterprise, 3066 North Carolina Central University, 1801 Fayetteville St., Durham, North Carolina 27707, United States

## Abstract

The development of sustainable smart packaging materials has driven interest in chitosan (CS) films, reinforced with conducting polymers. In the present work, poly­(1-naphthylamine) (PNA) was incorporated into CS matrix at low weight fractions (0.15–1.0 wt %) of PNA to produce composite films with enhanced mechanical and antibacterial performance. Characterizations using Fourier transform infrared, X-ray photoelectron, ultraviolet–visible, and fluorescence spectroscopy revealed significant interfacial interactions, as evidenced by distinct shifts in characteristic absorption peaks and the appearance of polaronic signatures. SEM and confocal imaging confirmed homogeneous PNA dispersion up to 0.5 wt %. The morphological transition from uniform network domains to the formation of agglomerates causes phase separation, which was noticed in composite films with higher PNA content (0.75 and 1 wt %). Mechanical testing indicated a substantial increase in tensile strength to 40 MPa for 0.15-PNA/CS compared to ∼5 MPa for pristine CS. The hybrid films exhibited reduced moisture uptake <20% and improved antibacterial activity against *Bacillus subtilis* , with zones of inhibition increasing from ∼0.9 cm for CS to 1.5–2.0 cm for PNA/CS composites. Molecular docking studies supported these findings, showing PNA bound strongly to bacterial target proteins, with the highest affinity (−10.0 kcal mol^–1^) at cavity 3 involving residues HIS353, GLU352, ASP339, and ARG364. Additional binding at cavities 1 and 2 (−7.9 and −7.0 kcal mol^–1^) suggests multisite inhibition of bacterial function. Collectively, these results demonstrate that PNA/CS hybrid films combine structural integrity, moisture resistance, and antimicrobial efficacy, making them promising candidates for active food packaging applications.

## Graphical abstract

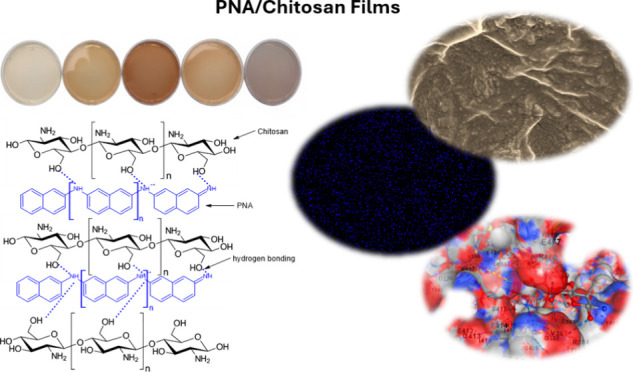

## Introduction

Growing environmental concerns over the use of synthetic plastics have accelerated efforts toward the development of sustainable and biodegradable alternatives for packaging material.^1^ Smart packaging materials are emerging as innovative solutions for enhancing food safety, extending shelf life, and providing real-time monitoring of food quality.^2,3^ Chitosan (CS), a natural biopolymer derived from chitin, has gained attention due to its biodegradability, antimicrobial activity, and film-forming ability. Despite its numerous advantages, pure CS exhibits several limitations that hinder its application as an independent packaging material capable of meeting commercial standards. These limitations include low mechanical strength, poor moisture resistance, and limited antimicrobial efficacy.^4,5^ The hydrophilic nature of CS causes it to readily take up moisture and swell upon exposure to humid environments, which significantly reduces its water vapor barrier efficiency and its capability to safeguard products sensitive to moisture.^6,7^ In addition, CS is polar in nature, which leads to poor compatibility with typical hydrophobic packaging polymers such as polyethylene (PE) and polypropylene (PP), making it necessary to employ compatibilizing agents/chemical additives to achieve uniform blending.^8,9^

To overcome these limitations, researchers have incorporated nanofillers, plasticizers, and biopolymers into CS matrices.^10,11^ Incorporating conducting polymers (CPs) such as polyaniline (PANI), polypyrrole (PPy), and poly­(3,4-ethylenedioxythiophene) (PEDOT) into packaging films has proven to be effective in designing smart, functional materials with enhanced barrier, antistatic, and sensing properties.^12–17^ CPs can impart improved moisture absorption, mechanical strength, UV resistance, and thermal stability, contributing to longer shelf life and better protection of packaged goods.^18,19^ Their tunable conductivity and compatibility with various substrates make them attractive for advanced, multifunctional packaging systems in food, pharmaceutical, and electronics industries.^20^

Poly­(1-naphthylamine) (PNA), a PANI derivative, has been explored for its unique electrical properties, chemical stability, and environmental responsiveness.^21,22^ PNA shows higher solubility as well as processability in solution than PANI and demonstrates an enhanced ability to form hydrogen bonds with chitosan (CS). Despite its immense potential, the incorporation of PNA into CS based packaging composite films remains unexplored. PNA, a conducting polymer, has not been previously studied in the context of smart packaging.^23,24^ This work attempts to bridge chemistry-focused film engineering with packaging applications. PNA can reduce moisture absorption by creating more compact, hydrophobic structures, thereby improving the shelf life and integrity of the moisture-sensitive products. PNA also exhibits intrinsic antibacterial properties and can even be functionalized with antimicrobial agents, providing active protection against microbial contamination.^25^ In the present work, PNA was introduced into the CS matrix through in situ chemical polymerization using varying weight ratios (0.15 to 1 wt %) of the former. This range of varying ratios was specifically chosen to attain homogeneous and well-dispersed composite films, as visual phase separation was noticed beyond 1 wt %. PNA loading, while concentrations lower than 0.15 wt % did not show any synergistic effect between CS and PNA to improve the physical properties of the composite films. The resulting composite films were then analyzed by using FTIR, UV–visible spectroscopy, XPS, and SEM techniques. Tensile strength, moisture absorption behavior, and antibacterial efficacy of PNA-CS composite films were thoroughly investigated to determine the potential of these composite films as functional packaging materials with enhanced durability, barrier performance, and microbial resistance. Complementing experimental antibacterial assays, molecular docking simulations with bacterial proteins were carried out to help identify the binding affinity, interaction sites, and possible mechanisms of inhibition, which support the development of more effective antibacterial materials.

## 2 Materials and Methods

Chitosan (CS, medium molecular weight ≈ 100 kDa, 85% degree of deacetylation, Fisher Scientific, USA), acetic acid (Fisher Scientific, USA), 1-naphthylamine (Fisher Scientific, USA), ethanol (Fisher Scientific, USA), anhydrous ferric chloride (Fisher Scientific, USA), and poly­(vinyl alcohol) (PVA, molar mass ≈ 10,000 Da, Fisher Scientific, USA) were used as received. LB broth with agar, Miller formulation (Fisher Bioreagents, USA), LB broth (Thermo Fisher Scientific, USA), and distilled water were also used without further purification.

### 2.1 Synthesis of CS Films

Chitosan powder (12.25 g) was dissolved in 900 mL of acetic acid and stirred magnetically at 95 °C for 24 h to produce a uniform, viscous solution. Separately, a 10% (w/v) poly­(vinyl alcohol) (PVA) solution was prepared by dissolving the polymer in 100 mL of deionized water. Following this, 100 mL of the CS solution (1.36 wt %) was combined with 1 mL of the 10% PVA solution, and the mixture was stirred until a consistent blend was achieved at 25 °C. A portion of the final solution was cast and left to dry at room temperature for 48 h.

### 2.2 Preparation of PNA/CS Composite Films

Varying masses of 1-naphthylamine (0.0214, 0.0358, 0.0715, 0.1073, and 0.1431 g) were each dissolved in 5 mL of ethanol at 25 °C. Ferric chloride (initiator) dissolved in (5 mL of ethanol) was added such that the molar ratio of monomer: initiator was maintained at 1:1. Each resulting solution was then added to 15 mL of chitosan (CS) solution and stirred on a magnetic stirrer for 2 h at 25 °C. The solution was left overnight, and after 24 h, when the polymerization was complete, excess ferric chloride was removed by centrifugation in deionized water and gentle agitation for 15 min. The rinse was discarded and replaced with fresh DI water; this cycle was repeated until the filtrate showed no visible coloration from Fe^3^^+^. The homogeneous suspensions were then poured into Petri dishes and allowed to dry under ambient conditions for 48 h, forming flexible composite films. This procedure was performed for all the different formulations, as shown in the table of Scheme 1b. The films were designated as 0.15-PNA/CS, 0.25-PNA/CS, 0.5-PNA/CS, 0.75-PNA/CS, and 1-PNA/CS based on loading of PNA. The chemical structure of PNA/CS composite films and the variation in color with loading of PNA are shown in Scheme 1a,b.

**Scheme 1:**
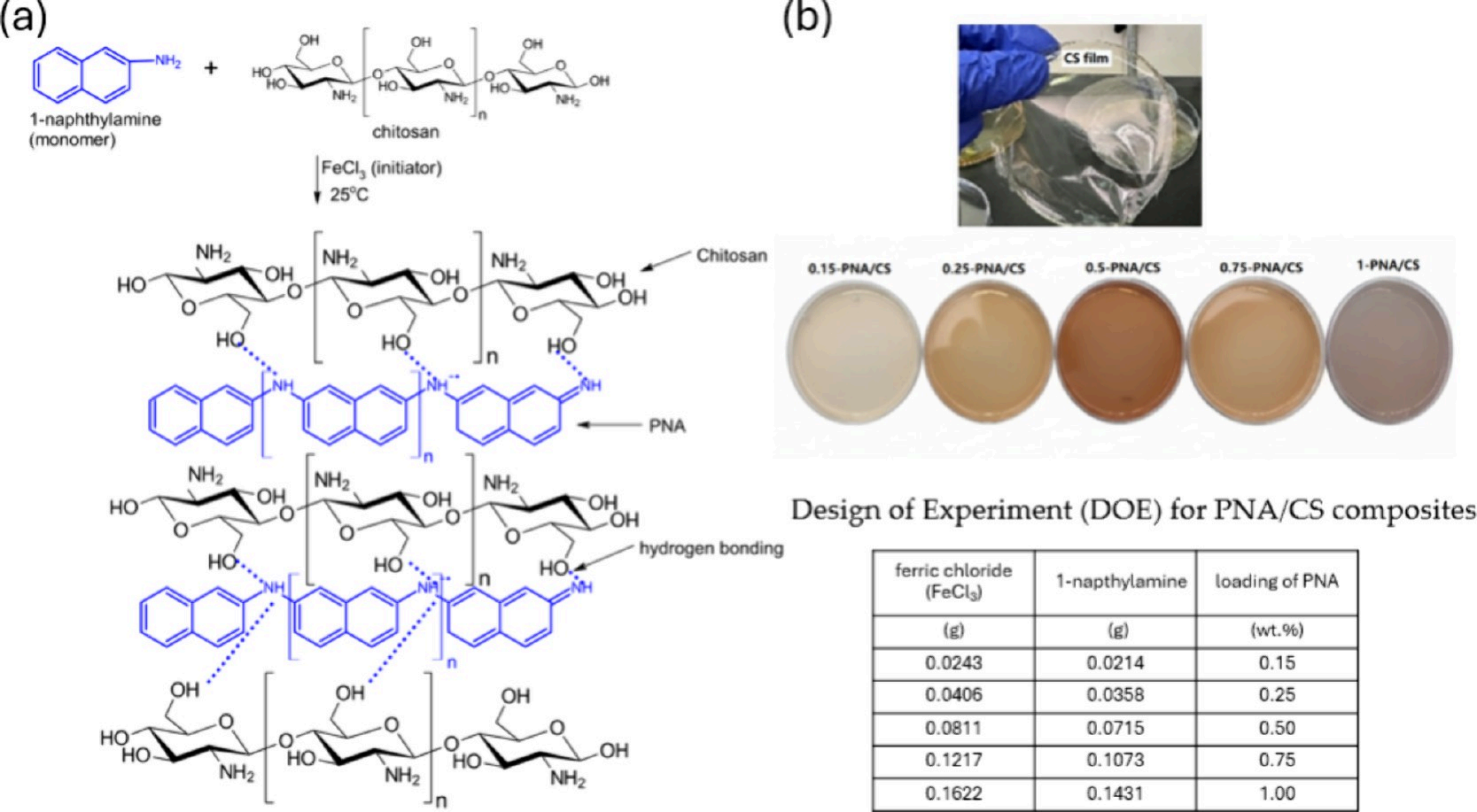
(a) Chemical Structure of PNA/CS Film, (b) Recipe for the Synthesis Showing Change in Color of Films with the Loading of PNA

## 3 Characterization

### 3.1 Spectral Analysis

Fourier transform infrared (FTIR) spectra of the PNA/chitosan (PNA/CS) composite films were obtained by using a PerkinElmer Spectrum Two FTIR spectrometer equipped with a diamond ATR (attenuated total reflectance) accessory. Measurements were carried out in the 4000–400 cm^–1^ range with a spectral resolution of 4 cm^–1^, and each spectrum was averaged over 30 scans to enhance signal quality. All analyses were performed at room temperature, and the resulting spectra were baseline-corrected and normalized prior to interpretation. The UV–visible absorption spectra of the PNA/Chitosan (PNA/CS) composite solutions were recorded using a PerkinElmer Lambda EZ 25 UV–Vis spectrophotometer with 1% (v/v) acetic acid serving as the blank solvent. PNA/CS samples containing various concentrations of PNA (ranging from 0.15 to 1 wt %) were prepared by dissolving 100 mg of the composite in 100 mL of 1% (v/v) acetic acid. The mixtures were magnetically stirred for 4–6 h at 30 °C to ensure complete dissolution and uniform dispersion of the components, yielding clear and homogeneous solutions. The absorption spectra were recorded over the wavelength range of 200–600 nm, and the corresponding λ_max_ values were determined to analyze the optical transitions and electronic interactions between PNA and chitosan. Fluorescence measurements of the PNA/chitosan (PNA/CS) composite solutions were performed using a Cary Eclipse fluorescence spectrophotometer. The samples were analyzed in the same solution form as prepared for the UV–visible experiments, maintaining identical solvent composition and concentration. The solutions were excited at 220 nm, and the corresponding emission spectra were recorded at room temperature to investigate the photoluminescence properties of the PNA/CS composites. XPS experiments were performed using a Physical Electronics Versa Probe III instrument equipped with a monochromatic Al kα X-ray source (hν = 1,486.6 eV) and a concentric hemispherical analyzer. Charge neutralization was carried out using both low-energy electrons (<5 eV) and Ar ions. The binding energy axis was calibrated using sputter-cleaned Cu (Cu 2p_3/2_ = 932.62 eV, Cu 3p_3/2_ = 75.1 eV) and Au foils (Au 4f_7/2_ = 83.96 eV. Measurements were made at a takeoff angle of 45° with respect to the sample surface plane. This resulted in a typical sampling depth of 3–6 nm (95% of the signal originated from this depth or shallower). Quantification was done by using instrumental relative sensitivity factors (RSFs) that account for the X-ray cross section and inelastic mean free path of the electrons.

### 3.2 Morphological Analysis

The surface morphology and elemental distribution of the samples were analyzed by using a scanning electron microscope (SEM) equipped with energy-dispersive X-ray (EDX) capabilities (FEI XL30 SEM-FEG). Confocal microscopy was performed using a Nikon A1R system integrated with a motorized inverted microscope (Model Ti-E), enabling both live-cell and spectral imaging.

### 3.3 Refractive Index and Moisture Absorption Studies

Refractive index (RI) measurements were carried out using an Abbey Refractometer at 28 °C. The moisture absorption equilibrium of the films was determined using water absorption (%) = *W*_h_ – *W*_i_/ *W*_i_ × 100, where *W*_h_ represents the hydrated weight of the film and *W*_i_ corresponds to the dry weight of the film. Four samples were used to replicate each condition.

### 3.4 Antibacterial Studies

The antibacterial activities of the samples were studied using a disc diffusion technique, where inhibition zones were observed. To make the composite samples into discs, a 5 mm diameter stainless steel puncher was pressed into films. Inhibition zone tests were performed on Gram-positive *Bacillus subtilis* ( *B. subtilis* ) cells. Briefly, freshly grown *B. subtilis* cells at an optical density (OD at 600 nm) of ∼0.55 were washed with PBS twice, and cells were resuspended and further diluted in PBS to an approximate OD of 0.016. Aliquots of 150 μL of the diluted cells were spread on LB agar plates and kept in the biological safety hood for 5–10 min before placing the composite film discs evenly spaced on agar plates. The plates were then incubated at 37 °C for 24 h. This procedure was replicated 5 times for each concentration (totaling 30 samples, including pure CS control). The diameter of the inhibition zones of each disc sample was measured after 24 h of incubation. ANOVA test was used to perform statistical analysis of the data to compare the antibacterial diameters of inhibition zones formed by the composite films. The two-sided significance level of *p* < 0.05 was used for assessing statistical significance.

### 3.5 Antioxidant Studies

Free radical scavenging activity was evaluated by using the 1,1-diphenyl-2-picrylhydrazyl (DPPH) assay. A stock solution was prepared by dissolving 2 mg of DPPH in 1 L of methanol. The resulting solution was filtered to obtain a working solution with an absorbance of approximately 0.973 at 517 nm. For the assay, 2 mL of the DPPH working solution was mixed with 10 μL of the PNA/CS sample in a test tube, followed by incubation in complete darkness for 30 min. The absorbance of the reaction mixture was then measured at 517 nm to determine the antioxidant activity. The following formula was used to compute the percentage of antioxidants:

$$\%\;\mathrm{of}\;\mathrm{antioxidant}\;\mathrm{activity}=\left[\left(A_{c}-A_{s}\right)÷A_{c}\right]\times 100$$

where *A*_c_ is the control reaction absorbance and *A*_s_ is the testing specimen absorbance. A linear plot of % inhibition versus concentration was analyzed using the equation of a straight line: *y* = *mx* + *c*, where *x* is the concentration of the measured substance and *y* is the % inhibition. Meanwhile, the IC_50_ value was determined as the *x* value of this equation when *y* was equal to 50%.

### 3.6 Docking Studies

The major housekeeping and stress-response protein in *B. subtilis* is the chaperonin GroEL (Hsp60), which is essential for protein folding and highly abundant under both normal and stress conditions. The docking studies were carried out to confirm the binding interaction of PNA/CS with the protein of *B. subtilis*. The 4DDQ protein was obtained from the Brookhaven Protein Data Bank (http://www.pdb.org) and was optimized by adding polar hydrogen followed by the addition of Kollman charges. After adding the ligand (PNA, CS, and PNA/CS), it was prepared by adding Gasteiger charges and saved as a pdbqt file. Autogrid parameter was used to generate a grid around the sites of the *Bacillus* protein with the ligand molecules. The molecular docking was then performed using the Lamarckian genetic algorithm. The binding site was chosen to suit *Bacillus* to allow the ligands to rotate freely. The binding position and conformation of the macromolecules with complexes and the rough estimate of their interactions were examined with the Auto Dock results. To analyze the mode of binding, the docked conformation with the lowest binding energy was selected.

## 4 Results and Discussion

### 4.1 Confirmation of Electrostatic Interactions via FTIR and XPS Studies

The functional groups and electrostatic interaction between PNA and CS were elucidated via FTIR studies (see the Supporting Information, Figure S1). In the neat CS spectrum, a broad absorption peak centered at ∼3294 cm^–1^ was assigned to NH/OH stretching vibrations of amine and hydroxyl groups of CS as per literature values.^26^ The peak at 1623 cm^–1^ corresponded to the C=O stretching of the acetyl moieties in CS. Introduction of PNA into the CS matrix induced a reduction in the peak intensity as well as a shift of the NH/OH peaks compared to pure CS, reflecting the formation of an electrostatic interaction via a hydrogen-bonding network as reported by other authors.^27^ In the 0.15-PANI/CS composite film, new peaks emerged around ∼1570 and ∼1091 cm^–1^, correlated to the characteristic C=C/C=N modes in PNA, while a peak at 648 cm^–1^ was attributed to PNA skeletal vibrations. At higher PNA loading (0.25-PANI/CS), additional peaks appeared at ∼1960 cm^–1^ (assigned to aliphatic chitosan segments), ∼1250 cm^–1^ (C–N stretching vibration of PNA), and ∼950 cm^–1^ (aromatic ring modes). In the 0.50-PANI/CS composite, these peaks persisted, and there was a minor shift of the PNA peak from ∼584 to ∼650 cm^–1^. Overall, the observed shifts and changes in intensity, especially in the NH/OH regions, support the formation of hydrogen bonds and electrostatic interactions between OH groups of CS and NH/NH_2_ groups of PNA. The decrease in intensity of the NH/OH peak was consistent with an increase in the loading of PNA, confirming higher interaction at higher loadings. Interestingly, the characteristic peaks of PNA showed no significant change with the loading. This indicates that the chemical structure and oxidation state of PNA remained essentially unchanged upon incorporation into the CS matrix. This stability also confirms that the interaction between PNA and CS is mainly physical, involving hydrogen bonding/electrostatic attraction, rather than chemical modification of PNA’s conjugated backbone. These intermolecular interactions play a significant role in the formation of a more integrated, uniform dispersion of polymer network, reduced phase separation, and improved mechanical as well as barrier properties, which are discussed in the upcoming sections.

X-ray photoelectron spectroscopy (XPS) was used to probe the surface chemistry and electronic interactions in the PNA/CS hybrid films. The high-resolution C 1s spectrum of the 0.15-PNA/CS composite, Figure 1a, showed peaks at ∼284.8 eV (attributed to C–H in CS), ∼286.3 eV (assigned to C–N in PNA quinonoid units), and ∼288.4 and ∼289.3 eV (corresponding to the O–C=O species from CS). The pronounced and broadened C–N signal implies a substantial presence of PNA at the surface. In the 0.25-PNA/CS composite film, Figure 1(b), the C–N peak shifted downward to ∼285.8 eV, and appeared to be broadened, as well as diminished in intensity. This behavior reflected stronger interfacial coupling in the local electron environment. Contributions from the O–C=O/C=O peaks diminished, consistent with increasingly PNA due to encapsulation of the CS oxygenated moieties. At higher PNA concentrations (0.50, 0.75), the trend was similar to that in lower loadings, Figure 1c,d, with the C–N peak area decreasing further, indicating progressive reorganization at the interface. Interestingly, in the 1.0-PNA/CS composite, Figure 1e, the C–N signal regained its intensity, suggesting microphase PNA aggregation and possible phase separation, which was confirmed by SEM analysis. Across all composite films, the relative attenuation of the O-containing peaks with rising PNA content confirmed that the surface becomes dominated by PNA, reflecting its strong interaction with the CS matrix.^28^ In the O 1s spectra, the 0.15-PNA/CS composite, Figure 1f, displayed peaks near ∼530.8 (C=O) and ∼532.7 eV (C–O). With increasing PNA content, Figure 1g–j, the signals shifted to higher binding energies (∼532.0 and ∼533.2 eV at 0.25 loading) and decreased in intensity, confirming modifications in the electron density around oxygen atoms, likely due to hydrogen bonding/electrostatic interactions with PNA.^28^ Beyond this loading, the intensity no longer drops significantly, implying that the surface chemical environment reaches a quasi-steady state as PNA coverage becomes dominant. The N 1s spectra (Figure 1k for 0.15-PNA/CS) revealed peaks at ∼398.8 (C–N) and ∼401.8 eV (protonated imine, C=N^+^) consistent with PNA’s chemical structure. With higher PNA incorporation, Figure 1l–o, the peak corresponding to C=N^+^ grew in relative intensity as well as in its area, which was indicative of an increasing population of oxidized quinoid/benzenoid units and enhanced protonation. This trend substantiates the progressive integration of PNA into the CS matrix and supports a model of increasing conjugation and electron delocalization in the hybrid films. These observations confirm the formation of a PNA network within the CS matrix, along with significant electronic and chemical interactions that contribute to the improved functional properties of the hybrid films.^27,28^ The increasing addition of π–π stacking, attributed to the alternating double single bonds of PNA addition, is a strong interaction, which reinforced the CS matrix and contributed to enhanced stability in forming network-type morphology within the composite structure. XPS showed reduced exposure of hydrophilic groups on the surface, which also corresponds to improved hydrophobicity with reduced water permeability of the composite films as seen in moisture absorption studies.

**Figure 1:**
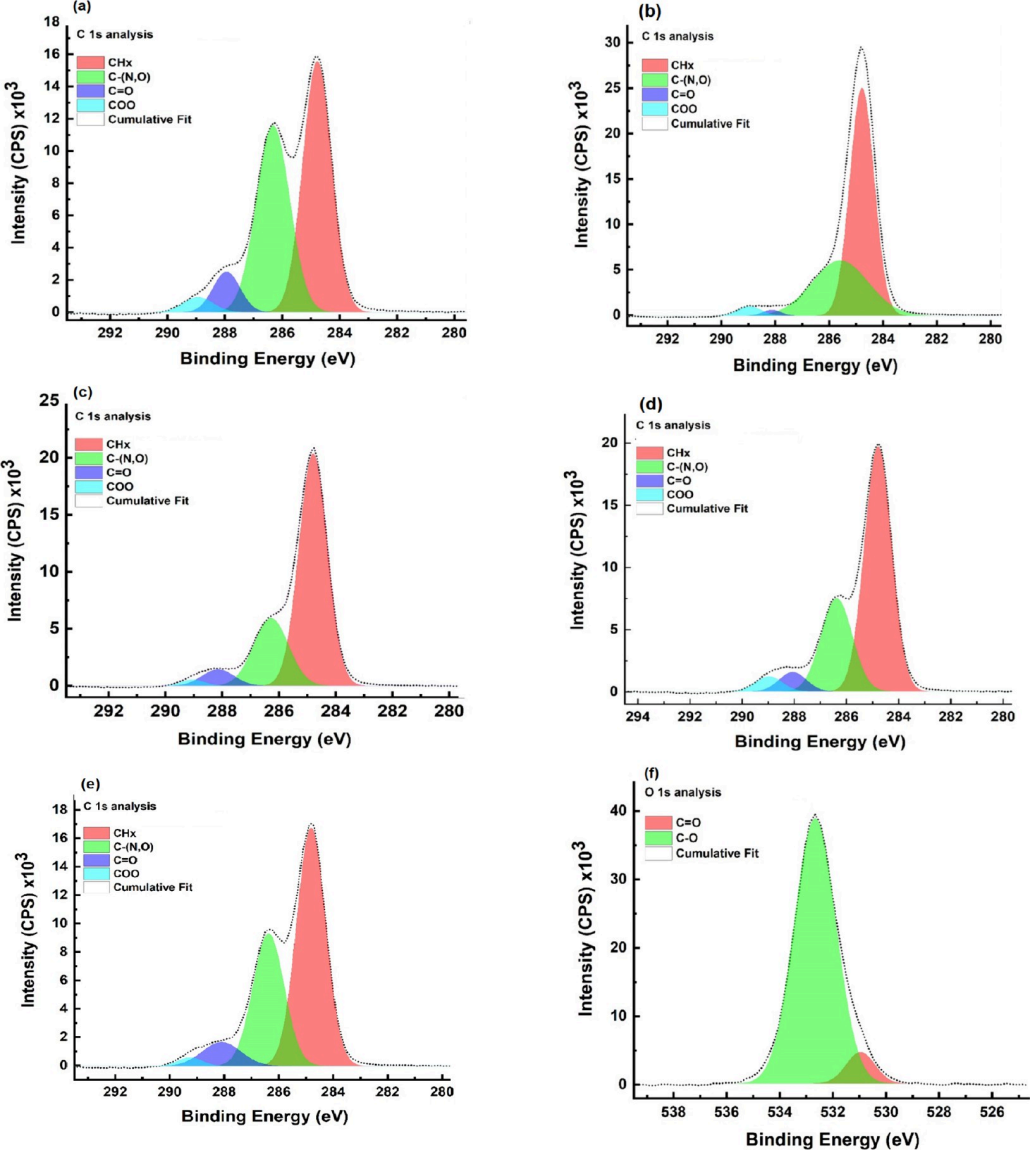

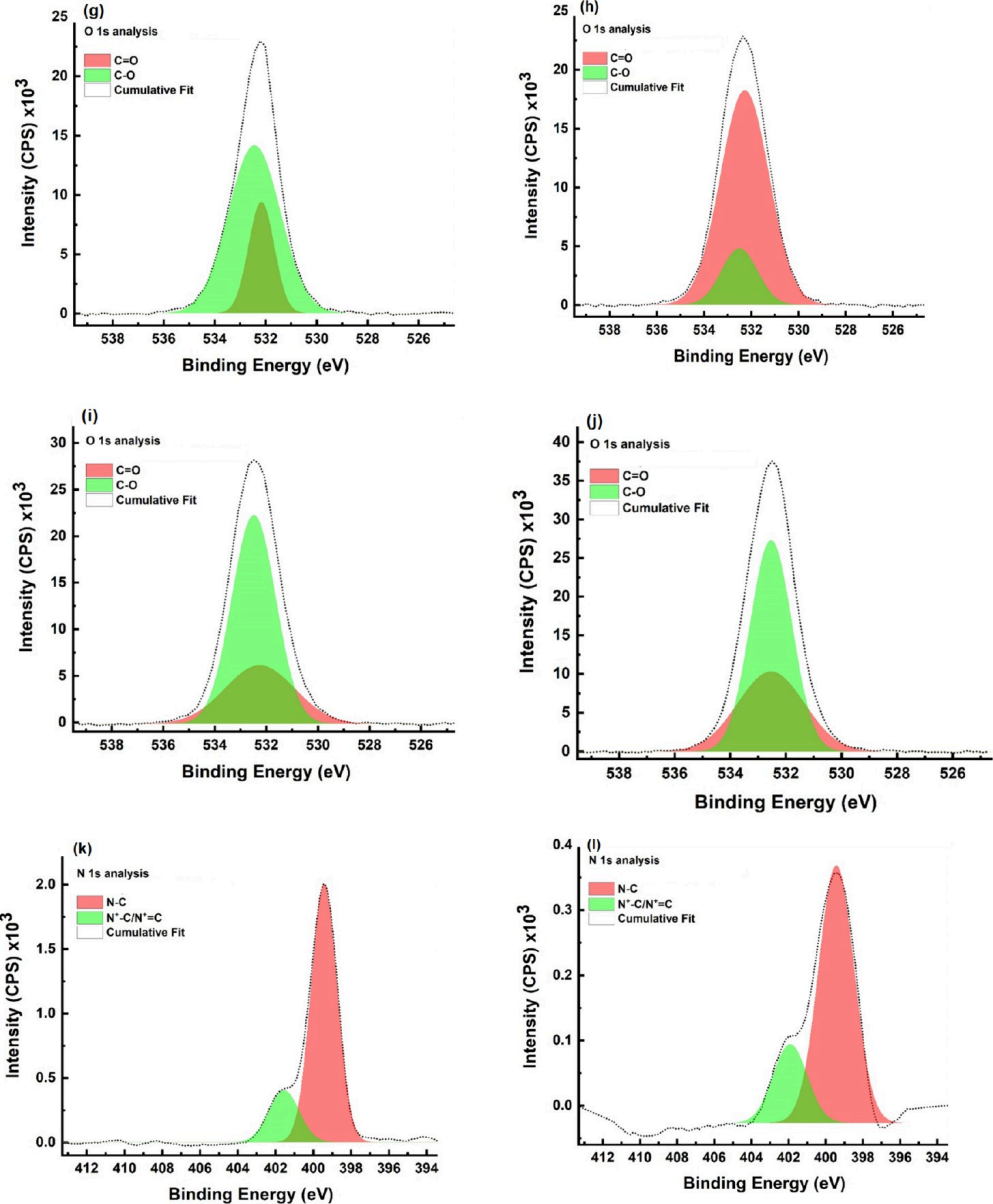

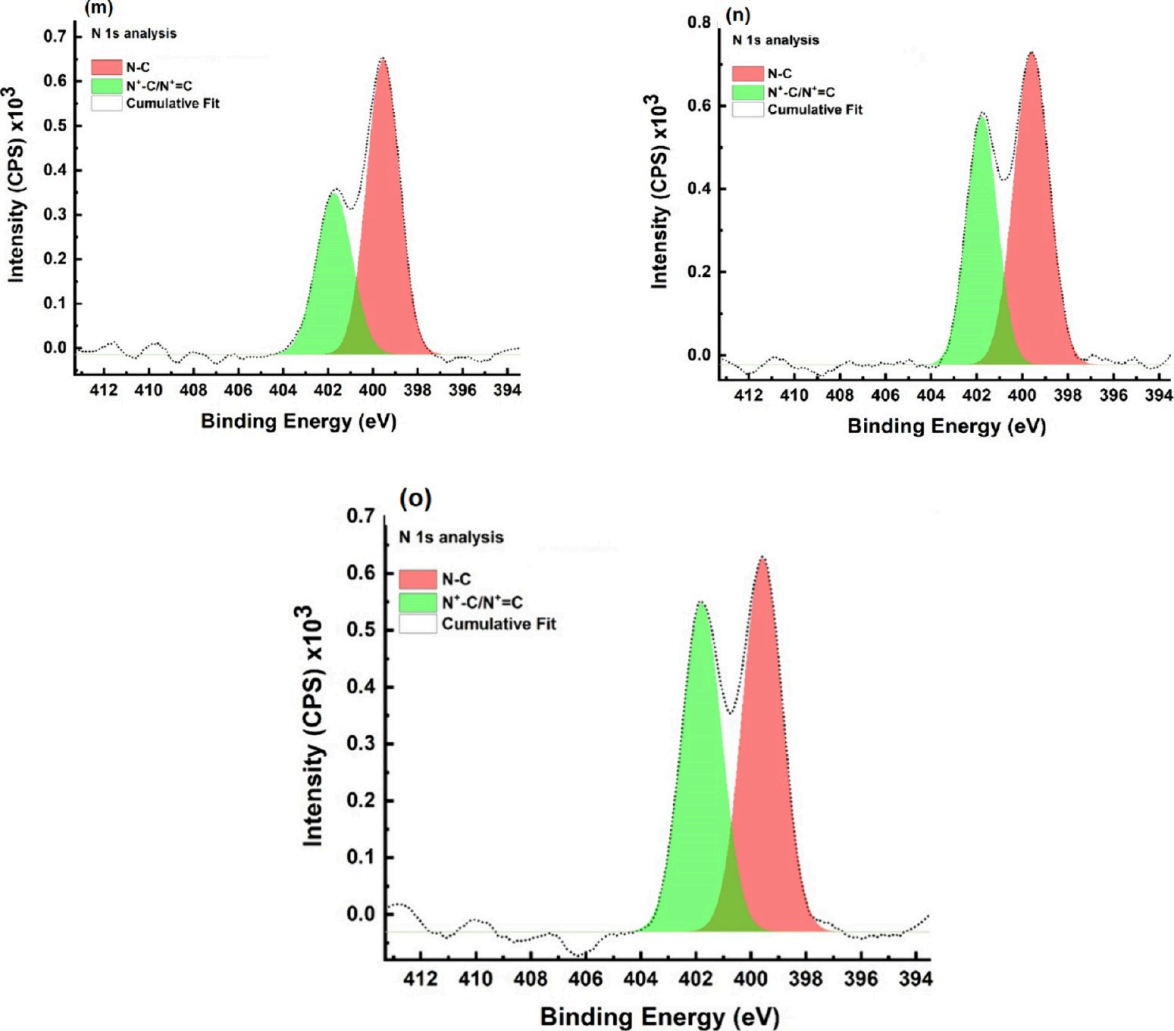
(a) C 1s spectrum of 0.15-PNA/CS, (b) C 1s spectrum of 0.25-PNA/CS, (c) C 1s spectrum of 0.5-PNA/CS, (d) C 1s spectrum of 0.75-PNA/CS, (e) C 1s spectrum of 1-PNA/CS, (f) O 1s spectrum of 0.15-PNA/CS, (g) O 1s spectrum of 0.25-PNA/CS, (h) O 1s spectrum of 0.5-PNA/CS, (i) O 1s spectrum of 0.75-PNA/CS, (j) O 1s spectrum of 1-PNA/CS, (k) N 1s spectrum of 0.15-PNA/CS, (l) N 1s spectrum of 0.25-PNA/CS, (m) N 1s spectrum of 5-PNA/CS, (n) N 1s spectrum of 0.75-PNA/CS, and (o) N 1s spectrum of 1-PNA/CS.

### 4.2 Changes in the Electronic Transition in PNA Confirmed via UV–Visible and Fluorescence Studies

The UV–visible spectra of PNA/CS films revealed distinct peaks at 280 and 510 nm, Figure 2a. The former peak was associated with π–π* transitions, while the latter peaks revealed polaronic transitions of PNA.^24^ Interestingly, the addition of PNA to the CS matrix showed a minor blue shift in the polaronic transition peak (around 550 nm), which was attributed to the electrostatic interaction between the OH groups of CS with the NH of PNA.^29^ The shifts in π–π* transitions in PNA when embedded in the CS matrix cause changes in the conjugation due to interactions such as electrostatic binding, affecting the electron density around the PNA chains.

**Figure 2:**
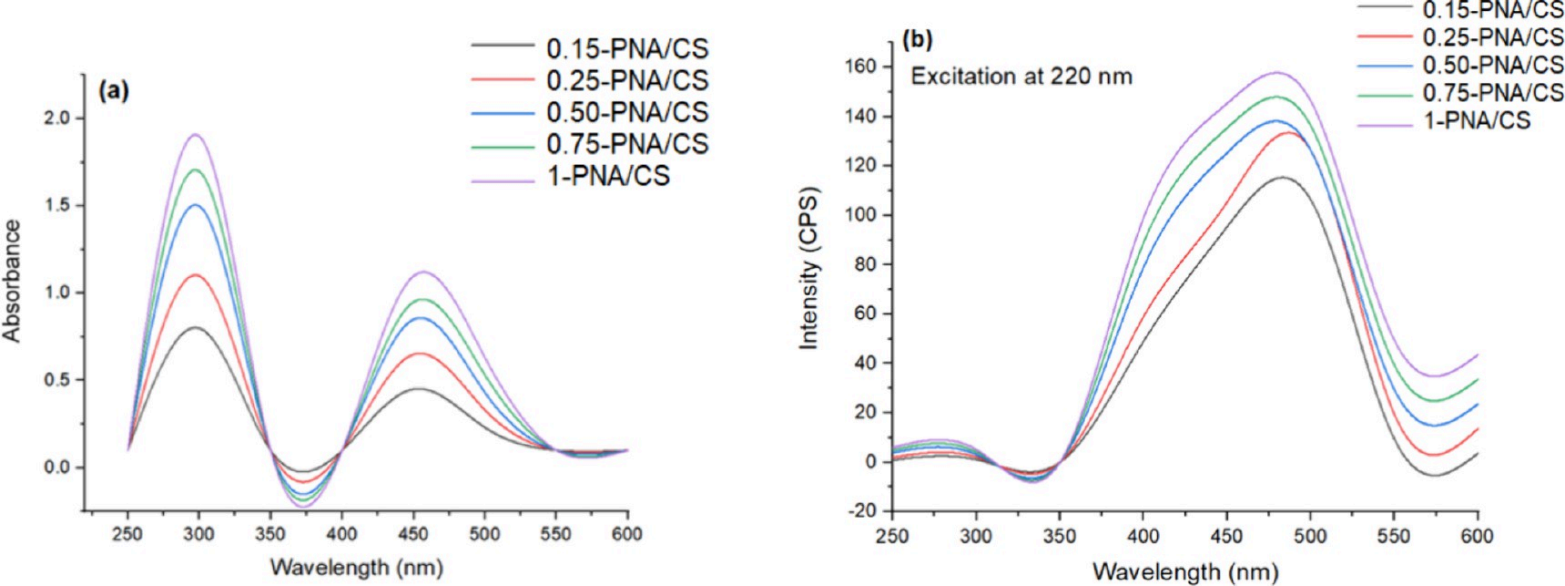
(a) UV–visible spectra of PNA/CS film hybrids. (b) Fluorescence spectra of PNA/CS hybrids.

The fluorescence spectra of the PNA/CS hybrid films, shown in Figure 2b, were recorded at an excitation wavelength of 220 nm. A broad emission peak centered around 470 nm was observed, with its intensity increasing proportionally with PNA loading in the CS matrix. This enhancement is attributed to π–π stacking and electrostatic interactions between PNA and the CS matrix.^30^ These interactions influenced the local electronic environment of PNA, leading to spectral broadening and a red shift in emission with an increase in its loading. These findings suggest that the incorporation of PNA significantly modulates the optoelectronic properties of the composite films. Confocal fluorescence imaging further supports these observations, revealing an enhanced and uniform fluorescence distribution across the film surface with higher PNA content.

### 4.3 Confocal Imaging Studies

Confocal fluorescence microscopy provides a detailed visualization of the spatial distribution of PNA within the chitosan (CS) matrix at various loading levels, where the observed intense blue emission originates from the PNA component. The confocal image of the 0.15-PNA/CS film, Figure 3a, revealed a highly scattered dispersion of PNA particles emitting in the blue region, consistent with the fluorescence spectrum showing a single emission peak at 470 nm.

**Figure 3:**
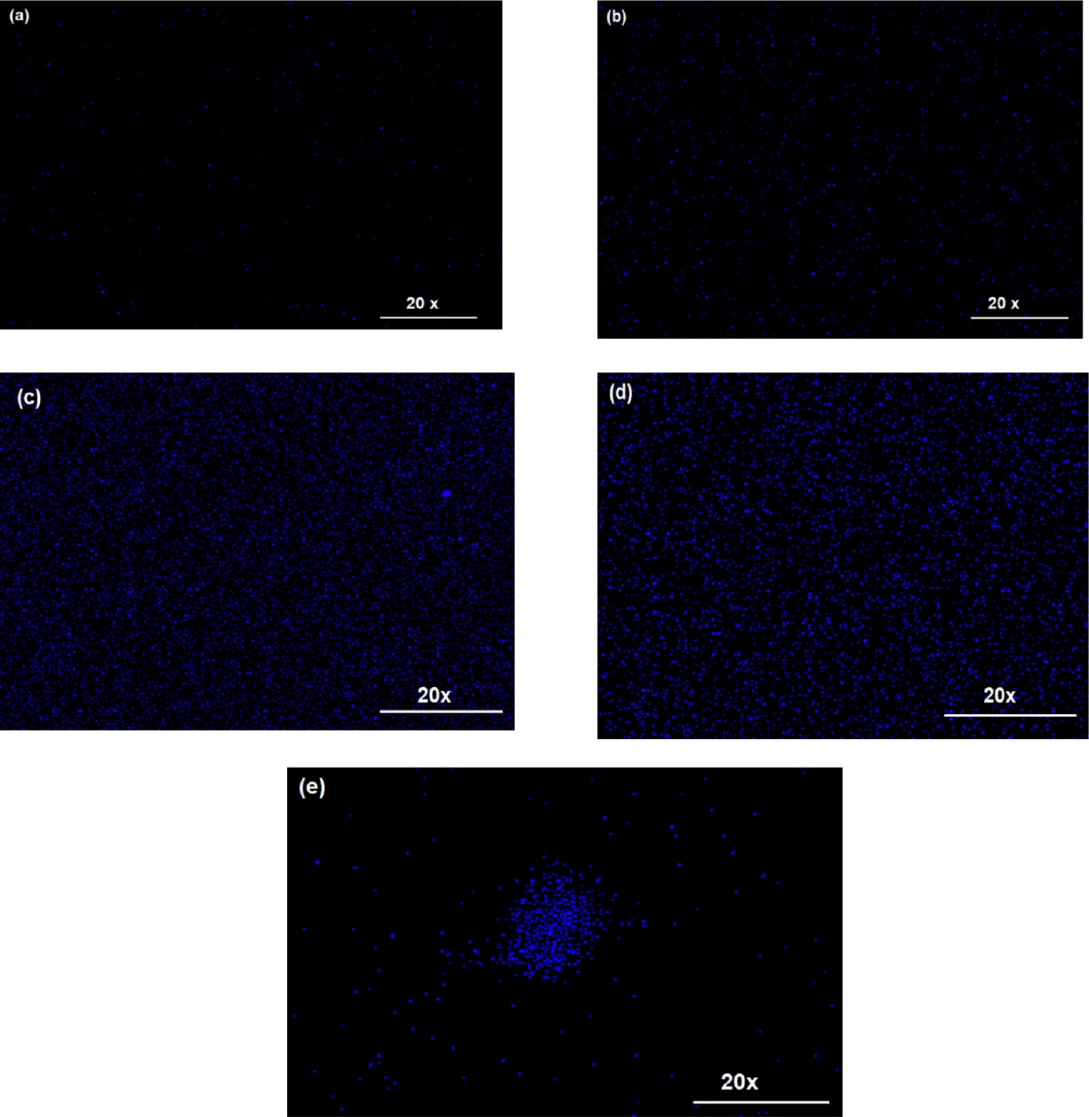
Confocal images of (a) 0.15-PNA/CS, (b) 0.25-PNA/CS, (c) 0. 5-PNA/CS (d) 0.75-PNA/CS, and (e) 1-PNA/CS hybrid films.

In contrast, the 0.25-PNA/CS sample and 0.5-PNA/CS, Figure 3b,c, displayed a more uniform dispersion of PNA particles with reduced scattering, suggesting improved distribution within the CS matrix. At higher PNA concentrations, 0.75-PNA and 1-PNA/CS (Figure 3d,e), the fluorescence images show increasingly dense dispersion of PNA domains and the onset of particle aggregation at this higher loading. These observations imply that PNA is well dispersed up to a critical concentration (0.5 wt %), beyond which phase separation/particle agglomeration begins to dominate. These findings are further corroborated by SEM analysis, which offers complementary evidence of the dispersion pattern of PNA within the composite films.

### 4.4 SEM Studies

Scanning electron microscopy (SEM) was utilized to examine the surface and cross-sectional morphologies of PNA/CS composite films prepared with different PNA loadings. The SEM micrograph of the neat CS film, Figure 4a, displayed smooth and homogeneous surface morphology, indicating a well-formed polymer matrix. The 0.15-PNA/CS composite film, Figure 4b, revealed fine white streaks associated with the presence of PNA embedded in the CS matrix. The corresponding fractured surface (given in the Supporting Information as Figure S2a) displayed a clear distinction between the white regions, attributed to PNA domains, and the gray areas corresponding to the CS matrix, confirming the formation of a composite structure. With increased PNA content (0.25-PNA/CS), the surface morphology (Figure 4c) showed the presence of distinct white domains associated with PNA, while the fractured surface (given in the Supporting Information as Figure S2b) presents a layered morphology, suggesting crust-like accumulation of PNA within the CS network.

**Figure 4:**
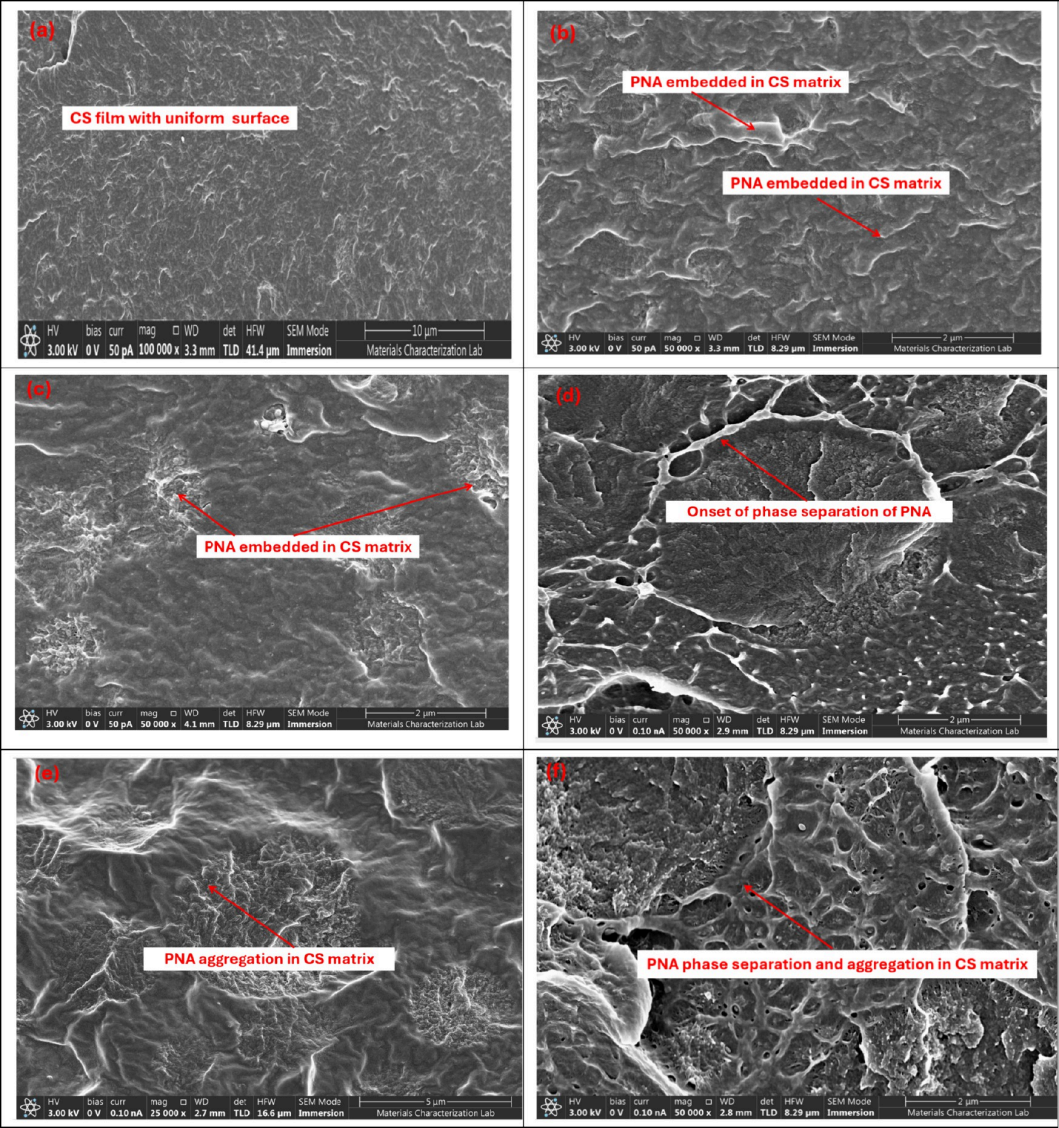
SEM images of (a) CS, (b) 0.15-PNA/CS, (c) 0.25-PNA/CS (d) 0.5-PNA, and (e) 0.75-PNA/CS (f) 1-PNA/CS.

As the PNA loading increases further to 0.5-PNA/CS, the surface SEM, Figure 4d, revealed network-like dispersion of aggregated PNA particles, while the cross section (given in the Supporting Information as Figure S2c) showed an aggregation of PNA to be distributed in a distinct layer adjacent to the CS phase. For the 0.75-PNA/CS film, SEM images (Figure 4e,h) exhibit a distinct dense and interconnected network of PNA embedded in the CS matrix, reflecting phase separation. The fractured morphology also confirmed aggregation (given in the Supporting Information as Figure S2d). At the highest loading in the case of 1.0-PNA/CS, the surface morphology, Figure 4f, was observed to be dominated by a clear phase-separated PNA network, revealing pores and intense aggregation of PNA chains. The fractured surface (given in the Supporting Information as Figure S2e) displayed a crust-like PNA layer overlaying the CS matrix, suggesting definite phase separation at elevated PNA concentrations. These morphological transitions, driven by increasing PNA content, reflect a progressive shift from uniformly dispersed embedded networked microstructural aggregation to finally phase-separated microstructural aggregation. At higher PNA concentrations, it is evident that agglomeration leads to reduced homogeneity. This roughened morphology at higher loadings also predicts poor mechanical strength of the composite films due to heterogeneous morphological transition and phase separation of PNA from the CS matrix. Such changes are expected to have a profound impact on the mechanical and moisture absorption properties of the composite films, which will be discussed in the upcoming section.

### 4.5 Influence of PNA Loading on Moisture Absorption, Refractive Index, and Mechanical Properties of PNA/CS Composite Films

The refractive index (RI) measurements of the PNA/CS hybrid films, as shown in Figure 5a, exhibited a slight decrease in the RI values with increasing PNA content. The RI value of neat CS film was observed to be 1.33, which is consistent with literature values.^31^ The increase in PNA loading up to 1 wt % showed no major reduction in the RI values, implying that strong electrostatic interactions between PNA and CS did not alter the electronic polarizability of the composite film and that the concentration was insufficient to produce large-scale delocalization of PNA, causing disruption of optical properties. CS is known to absorb approximately 80% of its weight in moisture under ambient humidity conditions (60–80% relative humidity); however, the moisture absorption capacity of the hybrid films was significantly reduced with increasing PNA loading, as illustrated in Figure 5b.

**Figure 5:**
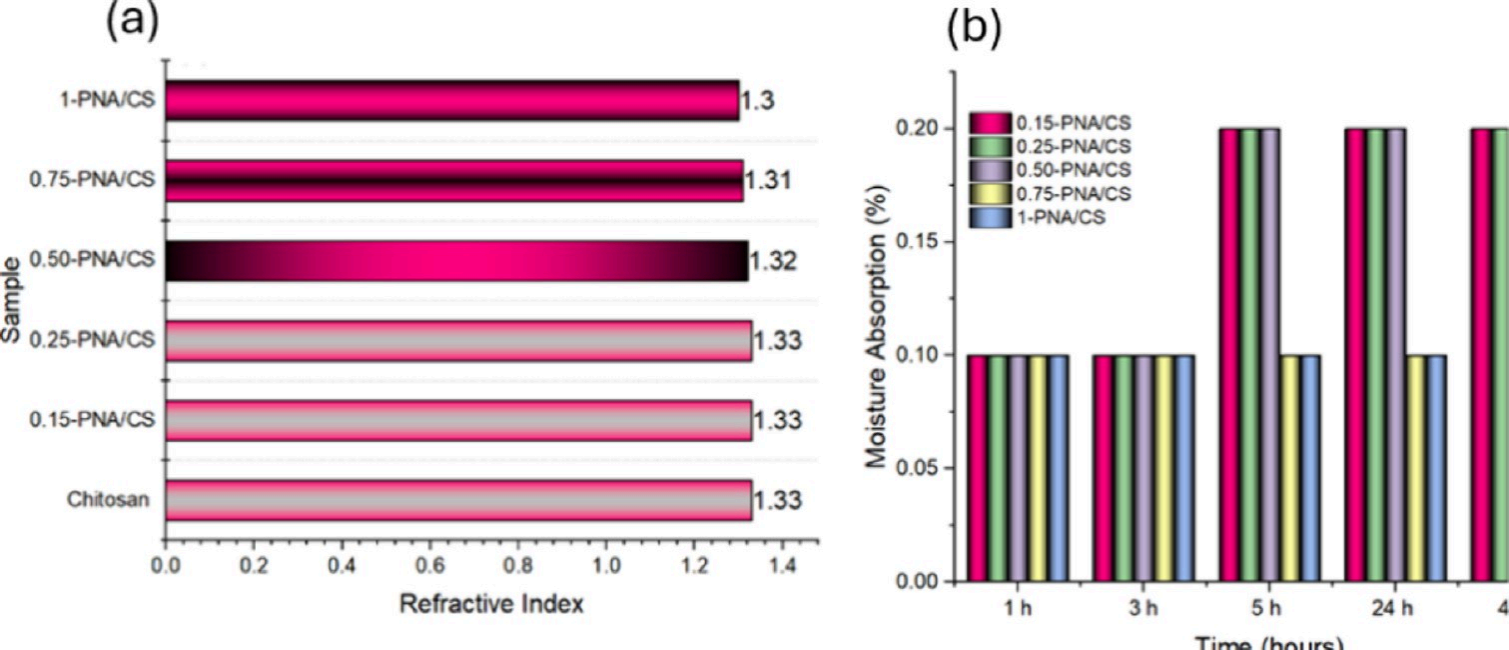
(a) Refractive index of PNA/CS films and (b) moisture absorption studies of PNA/CS films.

It was observed that the composite films of 0.75-PNA/CS and 1-PNA/CS showed 0.10% moisture absorption, which was consistent up to 48 h. However, the composite films of 0.15-PNA/CS, 0.25-PNA/CS, and 0.5-PNA/CS films revealed 0.10% moisture absorption up to 3 h, which increased to 0.2% upon 5 h exposure and remained constant up to 48 h. The moisture absorption was found to be significantly lower than neat chitosan, which absorbs 100% after 48 h in our case and 2415.5 ± 215.4% after 72 h as reported by other authors.^32^ The incorporation of PNA effectively lowers the hydrophilicity of the CS matrix, likely due to enhanced electrostatic interactions and formation of network-like structure with PNA which reduces the availability of free OH groups of CS for water binding. The reduced moisture absorption and swelling can be correlated to hydrogen bonding and covalent interactions between PNA and CS matrix that remarkably reduced the moisture absorption even upon loading of PNA as low as 1 wt %. Hence, the chemical modification of CS with PNA offers a promising strategy to tailor the moisture uptake behavior of PNA/CS composite films.

Furthermore, tensile strength (TS) properties of the films, presented in Figure 6a–c, demonstrated marked variations with different PNA loadings, highlighting the mechanical reinforcement effect imparted by PNA. The characteristics also revealed a drastic change upon varying the loading of PNA, Figure 6a. Pure CS exhibited relatively low TS (∼5 MPa), owing to its inherent brittleness and limited interchain interactions. Remarkably, the incorporation of a small amount of PNA for the 0.15-PNA/CS composite film significantly enhanced the TS value to approximately 40 MPa. This sharp increase is likely due to strong intermolecular interactions—particularly hydrogen bonding and π–π stacking—between PNA and CS matrix, which improved load transfer and network integrity as evidenced by the SEM image. With the increase in PNA from 0.25 to 0.75 wt %, a gradual decline in TS values was observed. This occurs due to the fact that when the PNA content becomes higher, the electrostatic interactions reach saturation, leading to aggregation and defect formation in the composite film, eventually causing phase separation of PNA from the CS matrix. Although these films still exhibited improved TS values compared to neat CS, the decrease was attributed to the disruption of uniform dispersion caused by clustering of PNA, which limits efficient stress distribution. The lowest TS value of ∼13 MPa was recorded for the 1.0-PNA/CS film, likely resulting from phase separation of PNA creating brittle domains that compromise mechanical coherence. These results indicated that the 0.15-PNA/CS composite film was optimal for achieving a desirable balance between strength and structural compatibility. The strain-to-break % of PNA/CS composite films varied significantly with PNA loading, as shown in Figure 6b. Neat CS exhibited brittle behavior with a strain-to-break below 1%. Introducing PNA enhanced flexibility at low to intermediate concentrations. Pure CS films exhibited a very low strain-to-break value of ∼1%, but in the 0.15-PNA/CS film, a noticeable increase in strain is observed, suggesting that low PNA loading acts as a reinforcing component, causing improved polymer chain movement and improved flexibility. The maximum strain (∼13%) was noticed for the 0.25-PNA/Cs composite film, indicating a well-balanced interfacial interaction between the PNA and CS matrix. The strain-to-break decreased sharply beyond 0.25 wt % loading of PNA due to microstructural inhomogeneity, causing increased stress concentration sites, leading to premature failure and reduced flexibility of the composite films.

**Figure 6:**
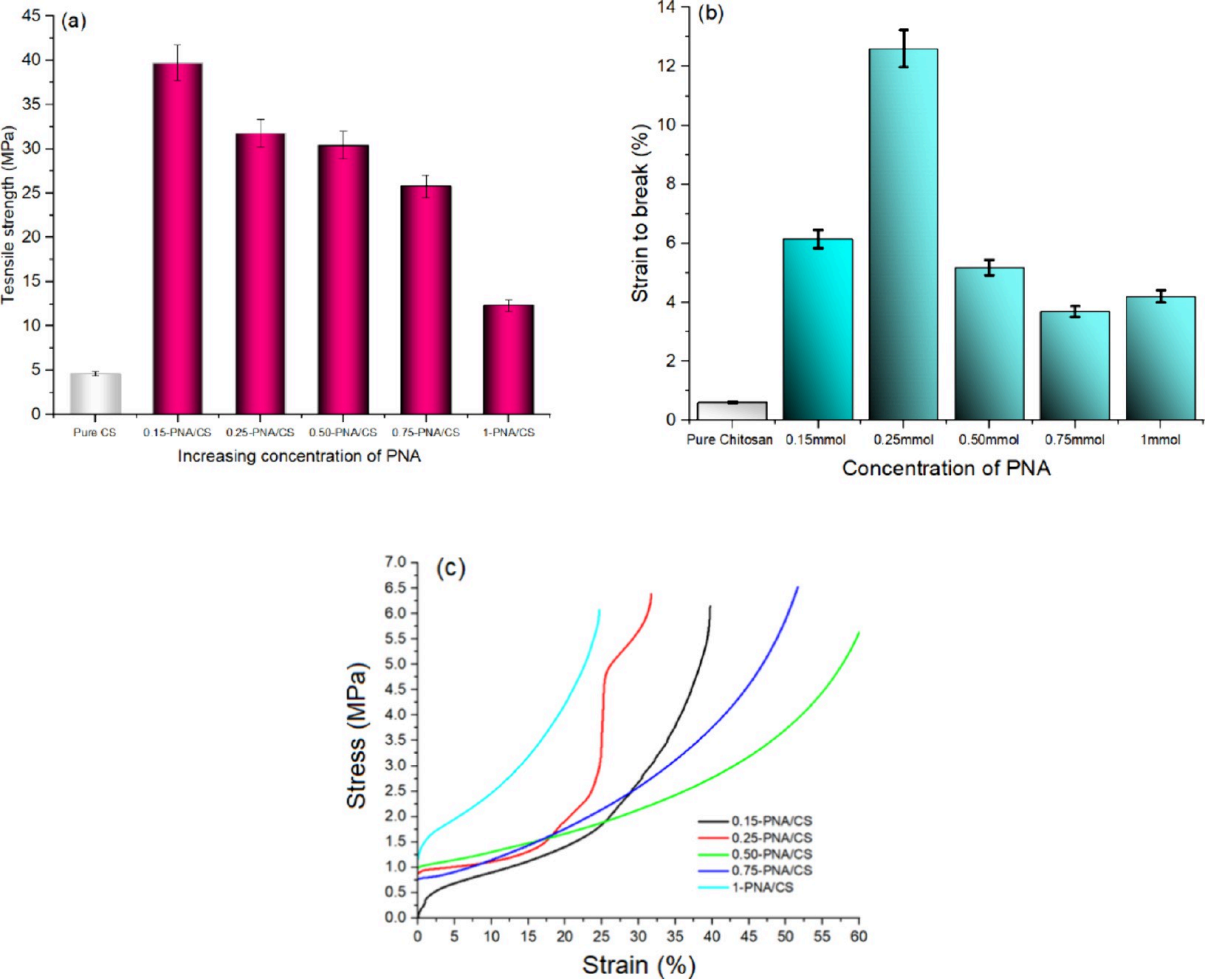
(a) Tensile strength of PNA/CS films, (b) strain-to-break profile of PNA/CS films, and (c) stress–strain curves of PNA/CS films.

The stress–strain curves of PNA/CS composite films shown in Figure 6c illustrate how the incorporation of varying concentrations of PNA influences the stress–strain behavior of the CS matrix. All samples exhibit an initial linear elastic region followed by gradual plastic deformation before fracture, characteristic of polymeric materials. Among the composite films, the 0.25-PNA/CS composite film demonstrated the highest stress and strain values, indicating superior TS and flexibility. The 0.15-PNA/CS composite film showed lower stress and strain values, and beyond 0.5 wt % concentration, both TS and strain decline progressively. The 0.75-PNA/CS and 1-PNA/CS composite films exhibit stiffer and brittle behavior, reflected by higher modulus values but reduced elongation at break. Although oxygen transmission rate (OTR) and water vapor transmission rate (WVTR) measurements are additional methods needed for assessing barrier properties in packaging applications, these tests are planned for future work to provide a comprehensive evaluation of the materials’ performance.

### 4.6 Variation in Antioxidant and Antimicrobial Activity upon PNA Loading in PNA/CS Composite Films

The antioxidant activity of PNA/CS films was measured using DPPH free radical assay (given in the Supporting Information as Figure S3a–e). After the incubation period of 1 h, a change in color from violet to yellow was noted, which confirmed the presence of antioxidant activity in PNA/CS composite film.^33–35^ Neat PNA showed a higher % inhibition than neat CS. Upon loading in the CS matrix, the % inhibition was found to be lower compared to neat CS and PNA due to electrostatic and hydrogen-bonding interactions between PNA and CS, which restrict the accessibility of active sites of PNA within the CS composite structure. For the composite films, the percent inhibition was overall found to increase with an increase in the loading of PNA, Figure 7a. The IC_5__0_ values were determined to quantify the radical quenching efficiency.

**Figure 7:**
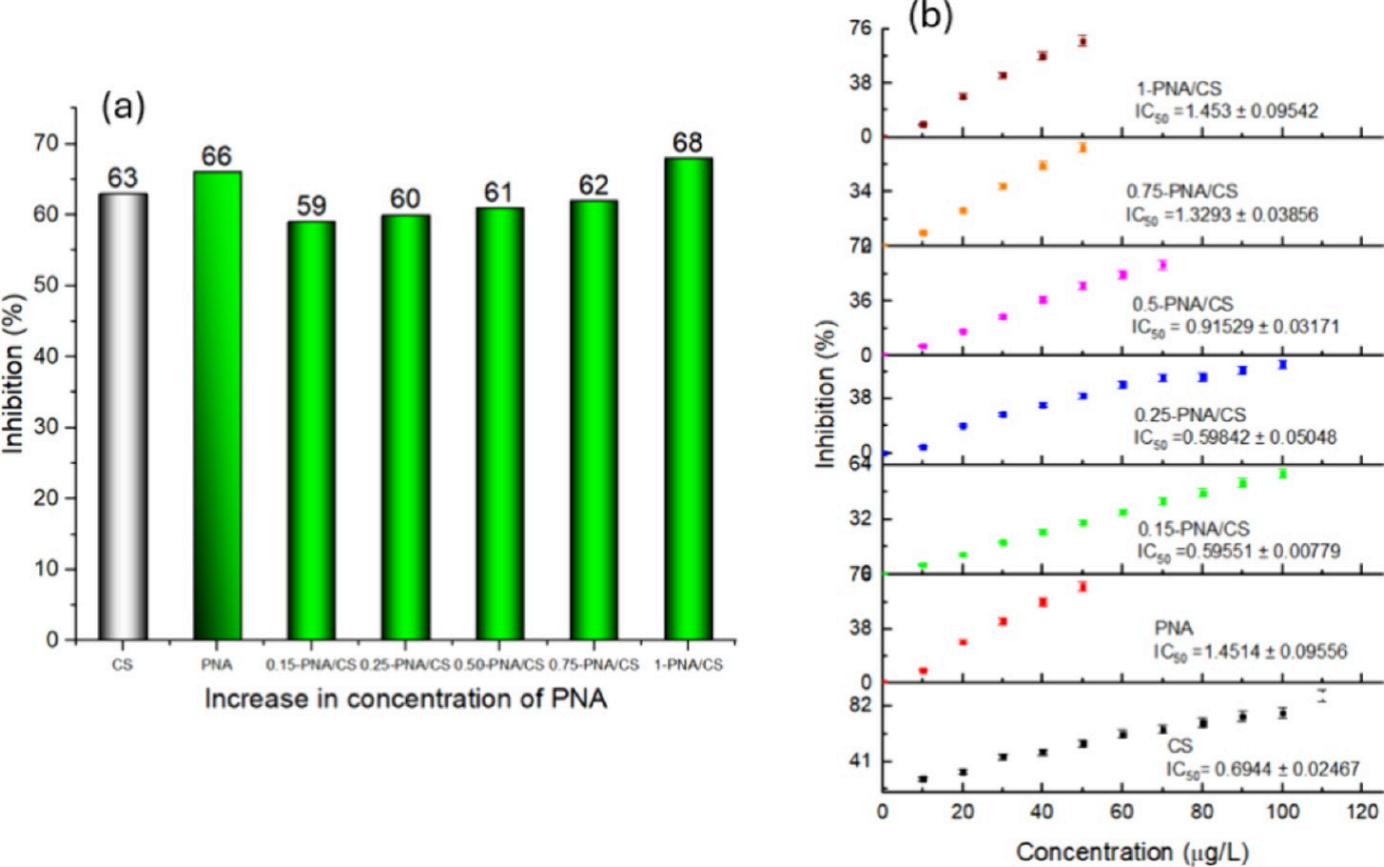
(a) Percent inhibition of CS/PNA and (b) IC_50_ curves of PNA/CS.

IC_50_ values were calculated based on the obtained linear regression graph, Figure 7b. Neat CS shows strong antioxidant activity as compared to neat PNA due to the availability of abundant hydroxyl and amino groups that participate in hydrogen atom transfer reactions, contributing to stronger radical scavenging efficiency. However, the composite films showed higher antioxidant activity as compared to neat PNA. Among all the composite films, 0.15-PNA/CS and 0.25-PNA/CS exhibited a strong antioxidant activity, with the lowest values in μg/mL.^35^ The values were found to increase beyond 0.25 wt % loading. A greater number of functional groups remain accessible within the CS matrix at lower PNA loadings, which effectively participate in electron/hydrogen donation, thereby enhancing antioxidant activity. As the PNA content increases up to 1 wt %, the accessibility of these active sites becomes restricted due to hydrogen bonding between the −NH groups of PNA and the −OH groups of CS, resulting in the reduced availability of free hydroxyl groups to participate in redox reactions. Consequently, the antioxidant performance of the nanocomposite films diminishes at higher PNA concentrations, despite the intrinsically electron-rich nature of PNA.

The antibacterial efficacy of the PNA/CS hybrid films was evaluated using the disc diffusion technique against *B. subtilis*, as shown in Figure 8a,b. Experimental trials were conducted to optimize the conditions and validate experimental methods. The data reported included five biological replicates per PNA concentration, totaling 30 samples (including pure CS control) for final statistics (given in the Supporting Information as Table S1). As shown in Figure 8a, a clear zone of inhibition (ZOI) was observed around each composite film disc on the agar plates, indicating the inhibitory effect of the composite films against *B. subtilis*. Higher concentrations of PNA loading showed a gradual increase in ZOIs. Composite films, CS and CS-PNA, were tested for antimicrobial affinity against Gram-positive *B. subtilis*. The results indicate that increasing PNA concentrations led to larger zones of inhibition, as shown in Figure 8(b). A one-way ANOVA was performed to determine whether the differences in ZOIs from the varying PNA concentration were statistically significant. The test revealed a significant effect of PNA on antibacterial activity with a *p*-value of 0.05. This supports that increasing the loading concentration of PNA into the CS matrix led to measurable and statistically significant improvements in the inhibition zones.

**Figure 8:**
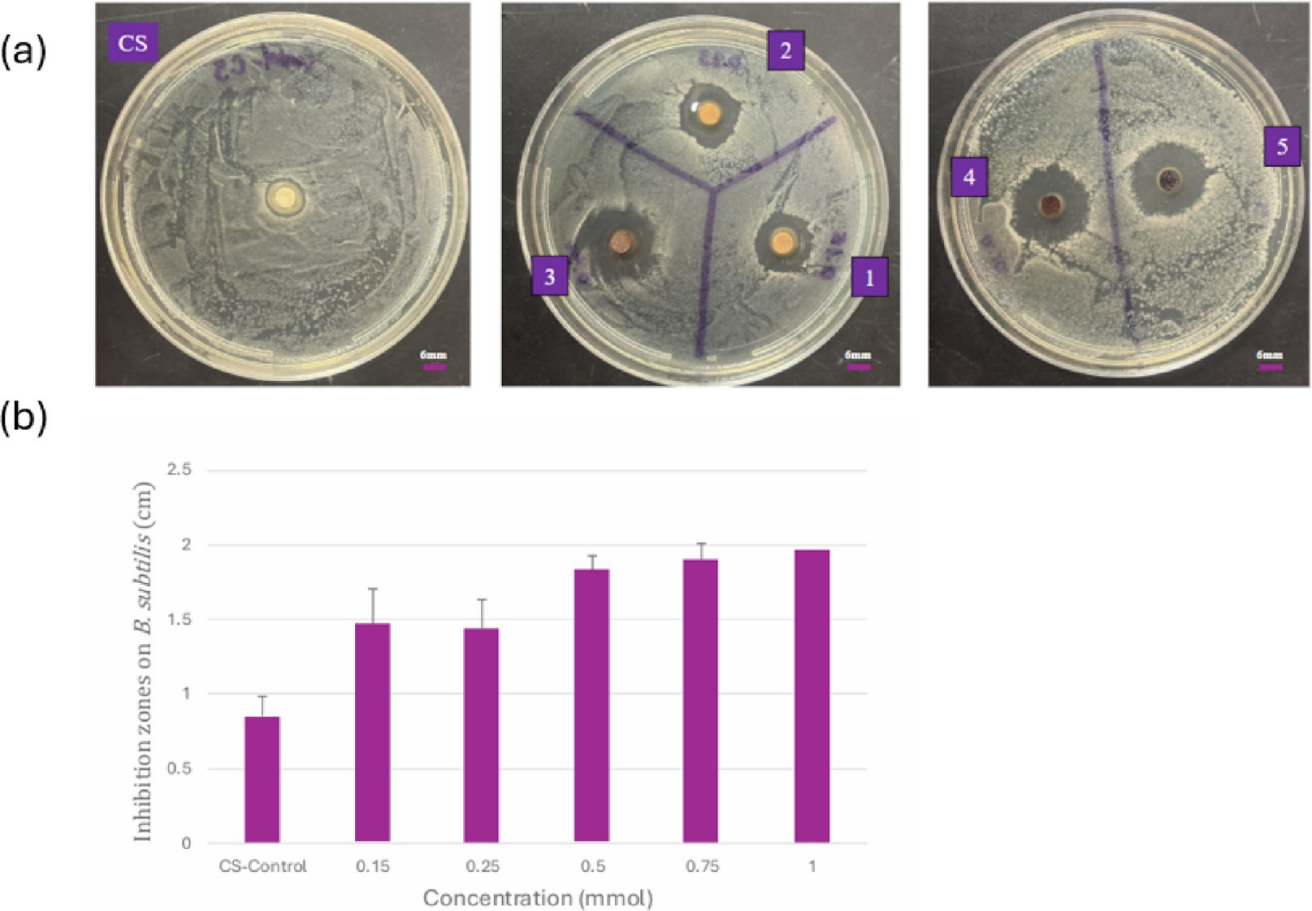
(a) CS: pure CS film (control); 1: 0.15-PNA/CS; 2: 0.25-PNA/CS; 3: 0.5-PNA/CS; 4: 0.75-PNA/CS; and 5: 1-PNA/CS; (b) zone of inhibitions (cm) for increasing PNA loading concentrations.

The molecular docking simulation presented illustrates the interaction between PNA-CS with *Bacillus subtilis* protein, Figure 9a,b, Table 1. The protein surface is depicted using electrostatic potential coloring, where red regions correspond to negatively charged (acidic) residues such as aspartic acid and glutamic acid, blue regions represent positively charged (basic) residues like lysine, arginine, and histidine, and white/gray areas indicate neutral or hydrophobic amino acids. This visualization enables the assessment of electrostatic complementarity between the ligand and the protein’s binding pocket. The ligand, shown as a ball-and-stick model, features aromatic rings, amine, and hydroxyl functional groups, which facilitate π–π stacking with aromatic residues (phenylalanine, tyrosine, and tryptophan), hydrogen bonding with polar residues (serine, threonine, asparagine, and glutamine), and electrostatic interactions with charged side chains.

**Figure 9:**
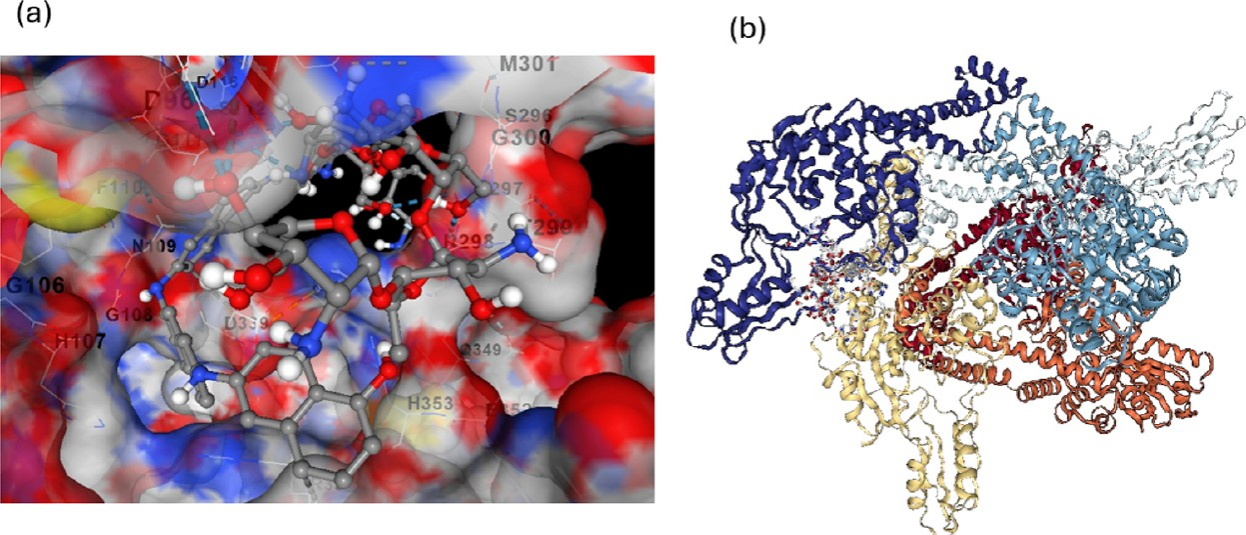
(a) 3D images of docking of *Bacillus subtilis* with (a) PNA-CS and (b) AutoDock Vina image of PNA-CS with *B. subtilis* protein.

**Table 1:** Summary of the Docked Cavity of Carbons (1–5) and Contact Sites of Protein with PNA/CS

ID	volume (Å^3^)	center coordinates (x, y, z)	Vina score	highlighted residues
1	3578	(−1.61, −62.51, −20.26)	–7.9	GLU374, GLU381, GLN420, TYR444, ARG478
2	3235	(−48.49, −42.45, −40.51)	–7.0	HIS378, GLU381, ARG384, TYR444, LYS455
3	2283	(8.31, −75.70, −43.72)	–10.0	HIS353, HIS357, GLY106, ASN109, ARG484
4	1567	(−59.73, −42.25, −13.13)	–7.5	HIS107, GLU125, TYR123, GLN147, PRO342
5	1093	(−58.14, −31.09, −16.68)	–7.4	HIS107, ASN109, ARG298, GLY340, LYS343

Key interacting residues such as H353 (His), D339 (Asp), S298 (Ser), and N109 (Asn) were positioned close to the ligand, indicating the formation of stabilizing noncovalent interactions, including hydrogen bonds and van der Waals forces. The binding pocket appears deep and well-defined, accommodating the ligand securely and suggesting a high binding affinity. These structural insights correlate well with fluorescence quenching studies, supporting the notion of static quenching through stable ground-state complex formation. Improvements in binding affinity and cavity interaction profiles were observed as compared to pure CS and 1-naphthylamine monomer (given in the Supporting Information as Figure S4 and Tables S2 and S3). Improved binding affinities and enriched interaction profiles were observed, with direct implications for the antibacterial potential of the ligand. Cavity 3 demonstrated the highest binding affinity with a Vina score of −10.0 kcal/mol, suggesting a strong and stable interaction with the target protein, Figure 9a,b. This cavity includes several key residues, such as HIS353, HIS357, GLU352, ARG364, and ASP339, which are often involved in enzymatic and regulatory functions essential for bacterial survival. Effective binding at this site could potentially inhibit critical processes such as cell wall biosynthesis and protein synthesis/energy metabolism, leading to bacteriostatic or bactericidal effects. Additionally, cavity 1 and cavity 2, with Vina scores of −7.9 and −7.0 kcal/mol, respectively, also revealed favorable interactions with functionally important residues like GLU381, TYR444, ARG478, and HIS107. These residues are a part of the active/allosteric sites involved in maintaining bacterial protein function. The repeated involvement of conserved residues across multiple high-affinity cavities suggests that the PNA/CS composite film shows the potential to disrupt essential bacterial functions at multiple targets. The strong binding affinity of −10.0 kcal/mol observed in docking cavity 3 of the *B. subtilis* protein is significant for antibacterial functionality because it indicates a highly stable and specific interaction between the ligand and a critical bacterial target site. A binding energy of this magnitude suggests that the PNA/CS forms multiple strong hydrogen bonds, hydrophobic contacts, and electrostatic attractions—with key amino acid residues essential for the protein’s biological activity. It also confirms that if cavity 3 corresponds to an active/regulatory site of an enzyme involved in vital bacterial processes (such as cell wall synthesis, metabolic regulation, or DNA replication), PNA/CS strong binding has the tendency to effectively block/disrupt that function, leading to growth suppression/apoptosis. Therefore, the strong docking interaction not only confirms molecular-level compatibility and stability but also implies that the composite film has the potential to target and deactivate protein sites of the bacillus.

## 5 Conclusions

This study demonstrates the successful fabrication of PNA-incorporated chitosan (PNA/CS) composite films with enhanced structural and functional attributes for smart and active packaging applications. The incorporation of PNA into the CS matrix resulted in significant improvements in the optoelectronic, mechanical, antioxidant, and antimicrobial properties. Spectroscopic and morphological analyses confirmed strong interfacial interactions and uniform PNA dispersion up to 0.75 wt %, beyond which aggregation led to reduced performance. Optimal mechanical strength and flexibility were observed at moderate PNA loadings (0.15–0.5 wt %), attributed to effective stress transfer and polymer chain compatibility. Antioxidant activity peaked at lower PNA concentrations (0.15–0.25 wt %) due to the availability of hydroxyl groups and favorable electron-donating effects. Molecular docking results further supported the antimicrobial potential of the hybrid films, with strong binding affinity observed between PNA and key residues within the DNA gyrase GyrA subunit of *B. subtilis* . Collectively, these findings highlight the potential of PNA/CS hybrid films as promising candidates for multifunctional packaging materials that combine sustainability with active and intelligent functionality.

## Supplementary Material



## Notes

Additional experimental and computational data that complement the main manuscript, including FTIR spectra and SEM images of PNA/chitosan (PNA/CS) hybrid films, UV–visible spectra for DPPH radical scavenging activity, 3D molecular docking images of *Bacillus subtilis* with PNA and chitosan, and detailed tabular data including ANOVA results and summaries of docking cavities and protein contact sites (PDF)
