# Supplementary material for: Poly(1-naphthylamine)-Reinforced Chitosan Films for Smart Packaging: Enhanced Mechanical, Morphological, and Antibacterial Properties
Source: Langmuir. 2025 Nov 12;41(46):31608–22. doi: 10.1021/acs.langmuir.5c04826 (PMC12659422; doi:10.1021/acs.langmuir.5c04826)
Supplement: Supplementary file 1 [file la5c04826_si_001.pdf]

Poly(1-naphthylamine)- Reinforced Chitosan Films for Smart Packaging: Enhanced Mechanical, Morphological, and Antibacterial Properties

Mary Taylor [1,2], Jayla Jenkins [1], Cristian Rodriguez[3], Audrey Adcock[3], Liju Yang[3], Mohammad Mohiuddin [2] and Ufana Riaz [1]*

[1] Biomedical/Biotechnology Research Institute, North Carolina Central University, Durham, NC, USA

[2] School of Packaging, Michigan State University, East Lansing, MI, USA

[3] Biomanufacturing Research Institute and Technology Enterprise, North Carolina Central University, Durham, NC, USA

*Correspondence: uriaz@nccu.edu

S.No	Figure/Table	Pg no
1.	Figure S1 FTIR spectra of PNA/CS hybrid films	S2
2.	Figure S2 SEM images of (a) CS (plain surface) (b) 0.15-PNA/CS (fractured surface), (c) 0.25-PNA/CS (plain surface), (d) 0.25-PNA/CS (fractured surface), (e) 0.5-PNA/CS (plain surface) , (f) 0.5-PNA/CS (fractured surface), (g) 0.75-PNA/CS (plain surface) (h) 0.75-PNA/CS (fractured surface), (i) 1-PNA/CS hybrid films (plain surface), (j) 1-PNA/CS (fractured surface)	S3
3.	Figure S3 UV-visible spectra of DPPH inhibition activity by (a) CS, (b) PNA, (c) 0.15-PNA/CS, (d) 0.25-PNA/CS, (e) 0.5-PNA/CS, (f) 0.75-PNA/CS, (g) 1-PNA/CS	S4
4.	Figure S4 3D images of docking of *bacillus subtilis* with (a) PNA, (b) CS	S6
5.	Table S1 ANOVA: Single Factor Results	S7
6.	Table S2 summary of the docked cavity of carbons (1-5) and contact sites of protein with CS	S7
7.	Table S3 summary of the docked cavity of carbons (1-5) and contact sites of protein with NAP	S8

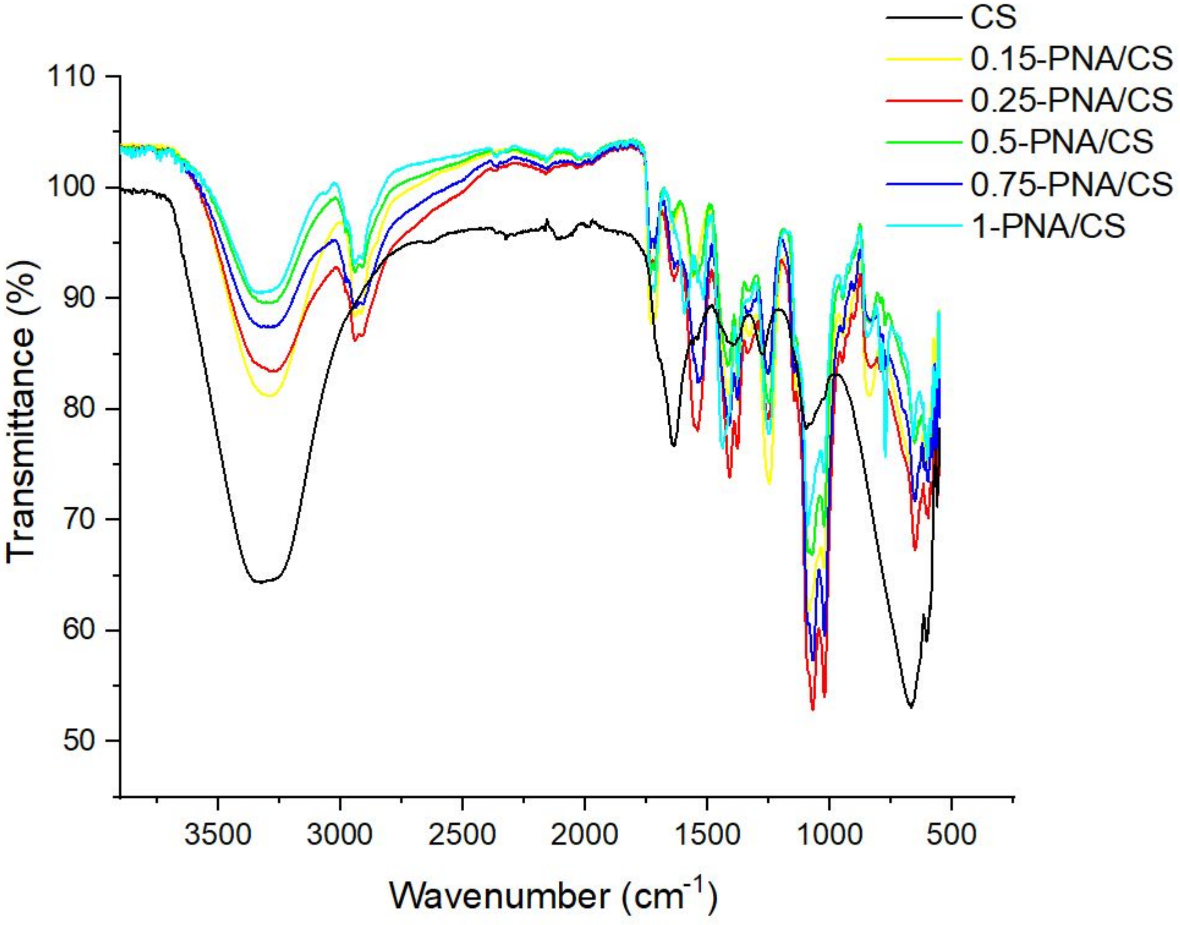

Figure S1 FTIR spectra of PNA/CS hybrid films

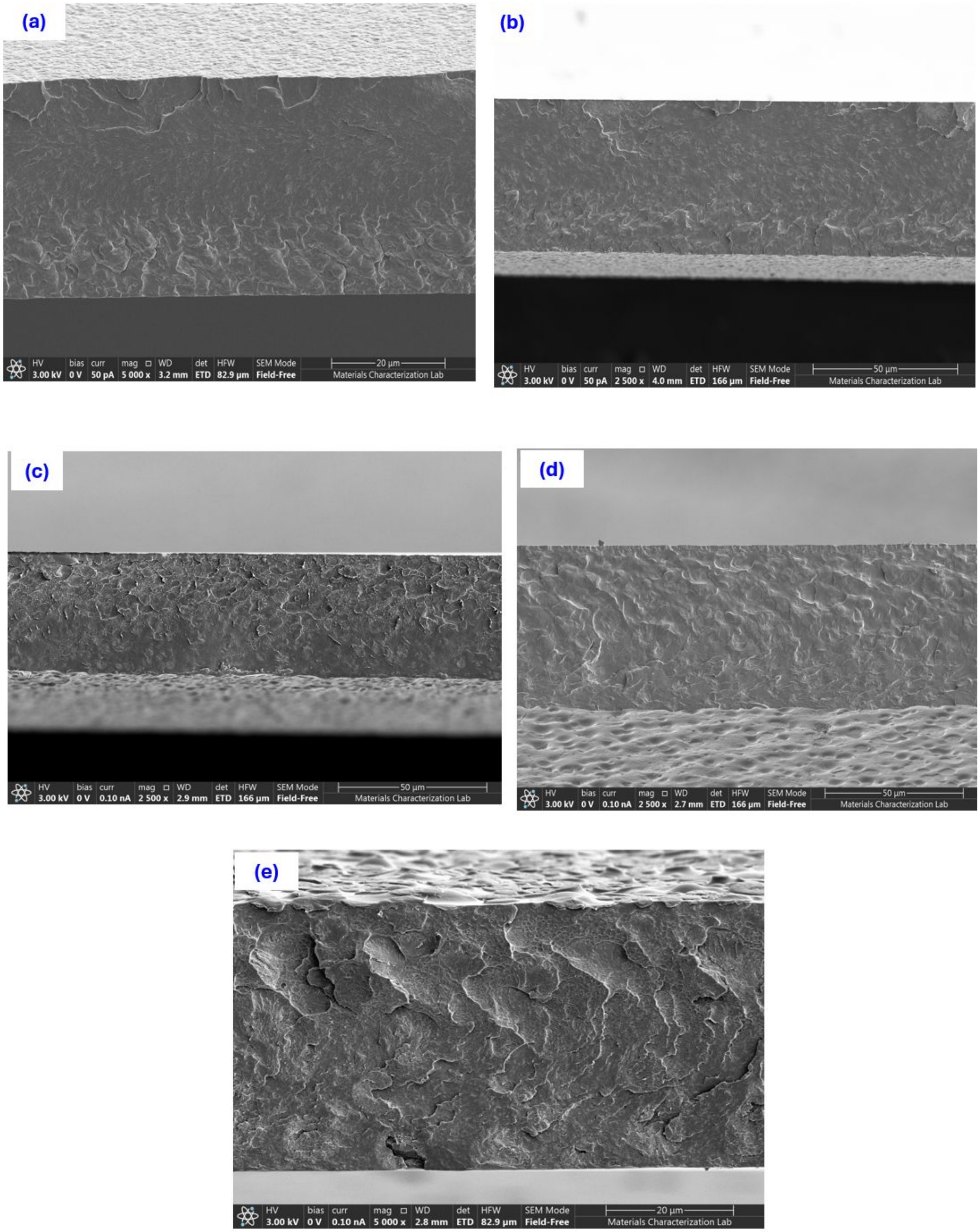

Figure S2 SEM images of fractured surfaces of composite films (a) 0.15-PNA/CS , (b) 0.25-PNA/CS, (c) 0.5-PNA/CS, (d) 0.75-PNA/CS (e) 1-PNA/CS hybrid films

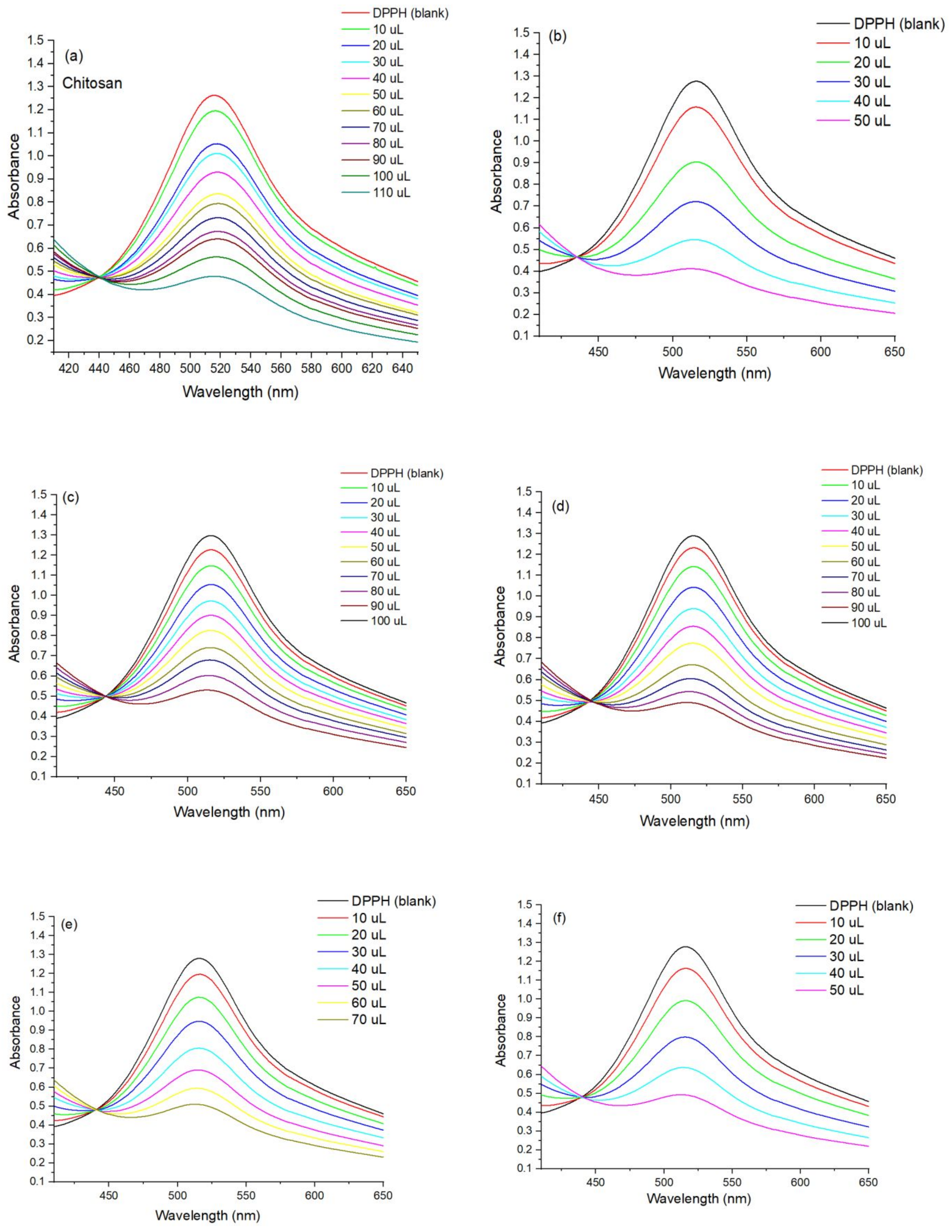
(a)
Chitosan
DPPH (blank)
10 uL
20 uL
30 uL
40 uL
50 uL
60 uL
70 uL
80 uL
90 uL
100 uL
110 uL
Absorbance
Wavelength (nm)
(b)
DPPH (blank)
10 uL
20 uL
30 uL
40 uL
50 uL
Absorbance
Wavelength (nm)
(c)
DPPH (blank)
10 uL
20 uL
30 uL
40 uL
50 uL
60 uL
70 uL
80 uL
90 uL
100 uL
Absorbance
Wavelength (nm)
(d)
DPPH (blank)
10 uL
20 uL
30 uL
40 uL
50 uL
60 uL
70 uL
80 uL
90 uL
100 uL
Absorbance
Wavelength (nm)
(e)
DPPH (blank)
10 uL
20 uL
30 uL
40 uL
50 uL
60 uL
70 uL
Absorbance
Wavelength (nm)
(f)
DPPH (blank)
10 uL
20 uL
30 uL
40 uL
50 uL
Absorbance
Wavelength (nm)

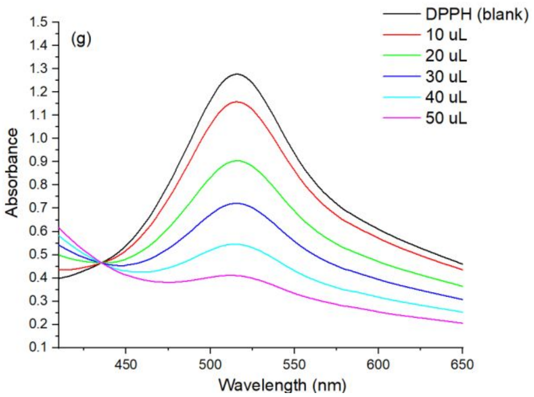

Figure S3 UV-visible spectra of DPPH inhibition activity by (a) CS, (b) PNA, (c) 0.15-PNA/CS, (d) 0.25-PNA/CS, (e) 0.5-PNA/CS, (f) 0.75-PNA/CS, (g) 1-PNA/CS

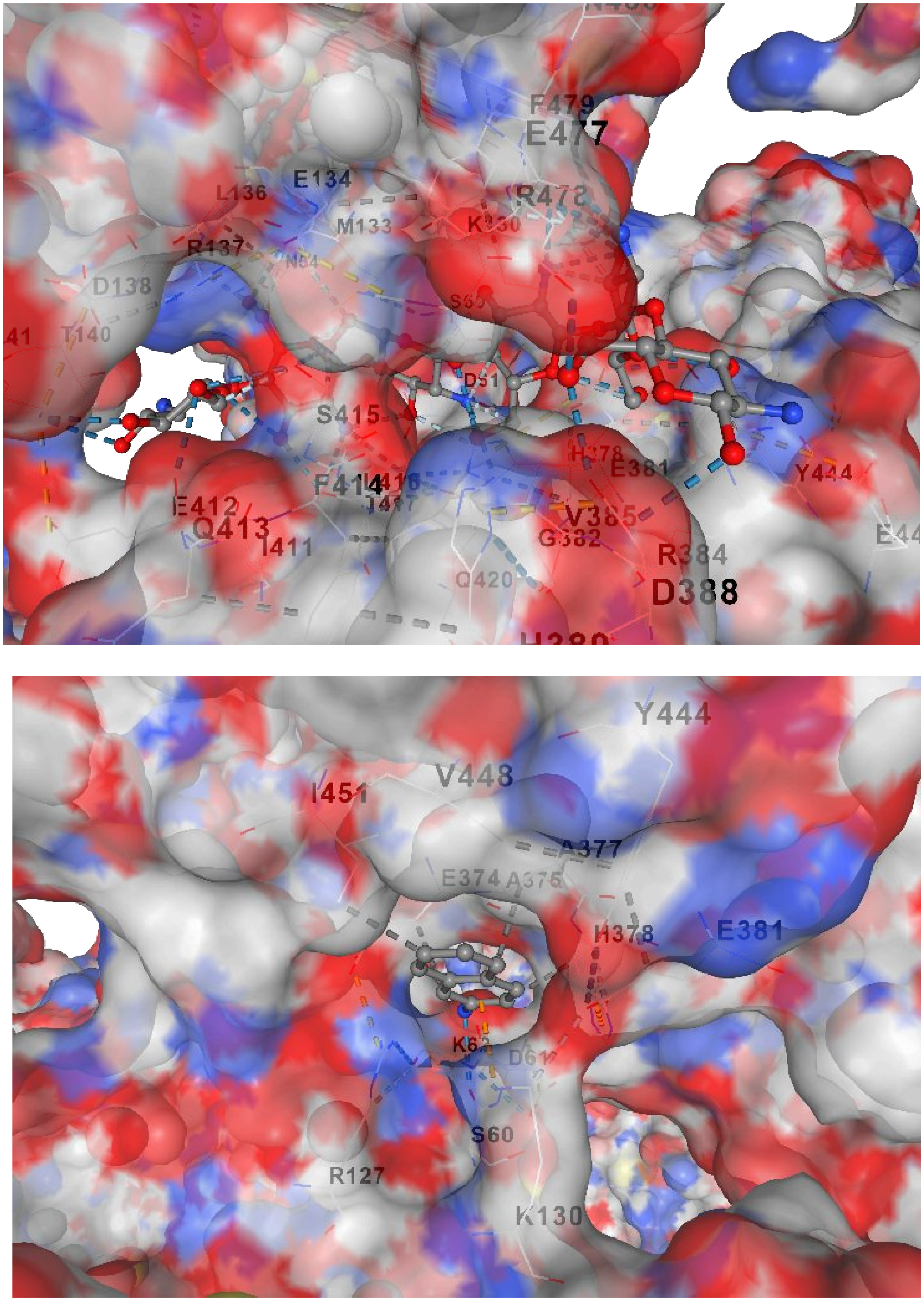

Figure S4 3D images of docking of *bacillus subtilis* with (a) PNA, (b) CS

Table S1 ANOVA: Single Factor Results

Groups	Count	Sum	Average	Variance		
0.15-PNA/CS	6	8.8	1.4666	0.0186		
0.25-PNA/CS	6	8.6	1.4333	0.05466		
0.5-PNA/CS	6	11	1.8333	0.03866		
0.75-PNA/CS	6	11.4	1.9	0.008		
1-PNA/CS	6	11.8	1.9666	0.01066		
**ANOVA**						
Source of Variation	*SS*	*df*	*MS*	*F*	***P-value***	*F crit*
Between Groups	1.5146	4	0.3786	14.4897	**2.9971E-06**	2.7587
Within Groups	0.6533	25	0.0261			
**Total**	2.1680	29				

Table S2 summary of the docked cavity of carbons (1-5) and contact sites of protein with CS

ID	Volume	Center (x,y,z)	Size (Å)	Score	Notable Contact Residues
1	3578	(-1.61, -62.51, -20.26)	37 × 37 × 37	-7.6	80+ residues; key ones include HIS378, ARG384, GLU412
2	3235	(-48.49, -42.45, -40.51)	37 × 37 × 37	**-8.5**	Strongest score; includes GLU134, HIS389, GLN420
3	2283	(8.31, -75.70, -43.72)	37 × 37 × 37	-8.2	ARG371, HIS353, ASP145 prominent
4	1567	(-59.73, -42.25, -13.13)	37 × 37 × 37	-7.6	Less volume; includes HIS107, GLU88, HIS79
5	1093	(-58.14, -31.09, -16.68)	37 × 37 × 37	-8.0	~100 residues; includes GLU275, HIS107, ARG127

Table S3 summary of the docked cavity of carbons (1-5) and contact sites of protein with NAP

**ID**	**Volume (Å³)**	**Center (x, y, z)**	**Size (Å)**	**Vina Score**	**Notable Contact Residues**
1	3578	(-1.61, -62.51, -20.26)	32 × 23 × 28	**-5.6**	GLU412, GLN413, PHE414, TYR444, ARG478
2	3235	(-48.49, -42.45, -40.51)	17 × 34 × 25	**-5.8**	GLU134, HIS378, PHE414, GLN420, ARG478
3	2283	(8.31, -75.70, -43.72)	25 × 26 × 17	-5.1	ASP339, GLN341, HIS353, GLU125, ARG298
4	1567	(-59.73, -42.25, -13.13)	34 × 17 × 17	-5.4	HIS107, GLU156, ARG122, TYR123, ARG47
5	1093	(-58.14, -31.09, -16.68)	17 × 17 × 17	-5.1	GLY106, HIS107, ASN109, HIS357, ARG364
